# Nitrogenase (*nifH*) gene expression in diazotrophic cyanobacteria in the Tropical North Atlantic in response to nutrient amendments

**DOI:** 10.3389/fmicb.2012.00386

**Published:** 2012-11-02

**Authors:** Kendra A. Turk-Kubo, Katherine M. Achilles, Tracy R. C. Serros, Mari Ochiai, Joseph P. Montoya, Jonathan P. Zehr

**Affiliations:** ^1^Department of Ocean Sciences, University of California at Santa CruzSanta Cruz, CA, USA; ^2^Southwest Fisheries Science Center, NOAA Fisheries ServiceLa Jolla, CA, USA; ^3^Center for Marine Environmental Studies, Ehime UniversityMatsuyama, Ehime, Japan; ^4^School of Biology, Georgia Institute of TechnologyAtlanta, GA, USA

**Keywords:** nitrogenase, P-limitation, Fe-limitation, UCYN-A, UCYN-B, *Trichodesmium*, diazotrophs, nitrogen fixation

## Abstract

The Tropical North Atlantic (TNAtl) plays a critical role in the marine nitrogen cycle, as it supports high rates of biological nitrogen (N_2_) fixation, yet it is unclear whether this process is limited by the availability of iron (Fe), phosphate (P) or is co-limited by both. In order to investigate the impact of nutrient limitation on the N_2_-fixing microorganisms (diazotrophs) in the TNAtl, trace metal clean nutrient amendment experiments were conducted, and the expression of nitrogenase (*nifH*) in cyanobacterial diazotrophs in response to the addition of Fe, P, or Fe+P was measured using quantitative PCR. To provide context, N_2_ fixation rates associated with the <10 μm community and diel *nifH* expression in natural cyanobacterial populations were measured. In the western TNAtl, *nifH* expression in *Crocosphaera*, *Trichodesmium*, and *Richelia* was stimulated by Fe and Fe+P additions, but not by P, implying that diazotrophs may be Fe-limited in this region. In the eastern TNAtl, *nifH* expression in unicellular cyanobacteria UCYN-A and *Crocosphaera* was stimulated by P, implying P-limitation. In equatorial waters, *nifH* expression in *Trichodesmium* was highest in Fe+P treatments, implying co-limitation in this region. Nutrient additions did not measurably stimulate N_2_ fixation rates in the <10 μm fraction in most of the experiments, even when upregulation of *nifH* expression was evident. These results demonstrate the utility of using gene expression to investigate the physiological state of natural populations of microorganisms, while underscoring the complexity of nutrient limitation on diazotrophy, and providing evidence that diazotroph populations are slow to respond to the addition of limiting nutrients and may be limited by different nutrients on basin-wide spatial scales. This has important implications for our current understanding of controls on N_2_ fixation in the TNAtl and may partially explain why it appears to be intermittently limited by Fe, P, or both.

## Introduction

Nitrogen (N_2_) fixation is an important component of the marine N_2_ cycle, as it serves as a source for biologically available N_2_ and can relieve N-limitation experienced by microbial communities living in oligotrophic regimes, in turn supporting a significant percentage of new production (Gruber and Galloway, 2008). High rates of biological N_2_ fixation (BNF) have been reported in the oligotrophic Tropical North Atlantic (TNAtl; Voss et al., 2004), and some estimates indicate that N_2_ fixation may support a large percentage of the export production in this region (Gruber and Sarmiento, 1997). Evidence indicates that these high BNF rates are influenced by Saharan dust deposition to surface waters in the TNAtl, which delivers dissolved iron (Fe) and to a lesser extent phosphorus (P; Mills et al., 2004), nutrients which are essential for the growth and activity microorganisms with the metabolic capability of N_2_ fixation, termed diazotrophs.

It remains unclear whether BNF in the TNAtl is ultimately limited by the availability of Fe, P or is co-limited by both. Several studies have argued that high fluxes of Fe-rich Saharan dust in to the TNAtl drive P-limitation of diazotrophy (Sanudo-Wilhelmy et al., 2001). More recently, Moore et al. (2009) correlate the different magnitudes of N_2_ fixation in the North and South Atlantic to Fe inputs rather than P availability, and ultimately argues that on large spatial scales, N_2_ fixation is limited by Fe in the Atlantic Ocean. However, direct experimental measurements of the response of N_2_ fixation to P or Fe additions in the TNAtl is limited to a study conducted by Mills et al. (2004), in which N_2_ fixation was only stimulated by the addition of both Fe and P, or by Saharan dust (presumed to have both) in the easternmost part of the basin.

A diverse community of diazotrophs has been described in this ocean basin that includes *Trichodesmium*, unicellular cyanobacteria (UCYN-A and *Crocosphaera*, also referred to as UCYN-B), and the heterocystous symbiont *Richelia* associated with diatoms *Rhizosolenia clevi* (sometimes abbreviated as Het-1, but will be referred to as RR herein) and *Hemiaulus hauckii* (Het-2, HR herein; Langlois et al., 2008; Foster et al., 2009; Goebel et al., 2010). Goebel et al. (2010) reported on the spatial and depth distribution of these cyanobacterial phylotypes across the TNAtl, determined using quantitative polymerase chain reaction (qPCR) assays targeting a gene in the nitrogenase operon, *nifH*, and used a subset of this *nifH*-based abundance data in a diagnostic model to predict contributions of several phylotypes to N_2_ fixation in these waters. *Trichodesmium* was the dominant diazotroph at most of the stations surveyed, and the resulting model suggests that it is also responsible for a majority of the N_2_ fixed in these waters. However, in the easternmost stations around the Cape Verde Islands, abundances of the uncultivated unicellular cyanobacteria, UCYN-A, exceeded those of *Trichodesmium*, suggesting that UCYN-A may be the most important contributor to N_2_ fixation rates in this region. A separate study in the vicinity of the Cape Verde Islands, reported that *nifH* expression in UCYN-A was consistently higher (per L of seawater) than in other diazotrophs, including *Trichodesmium* (Turk et al., 2011). Together with the studies of Langlois et al. (2008) there is an emerging pattern of distinct spatial variability of the dominant diazotrophs in the TNAtl. However, there is a paucity of data on the expression of *nifH* in these phylotypes throughout this basin, and more importantly, there have been no direct measurements of how additions of Fe and P to natural populations of diazotrophs in the TNAtl impact the expression of *nifH* in individual cyanobacterial phylotypes (which can be considered a proxy for active N_2_-fixing activity).

The biological reduction of N_2_ to biologically available N is energetically expensive and has high Fe requirements, due to the use of Fe as a cofactor in the nitrogenase enzyme. Furthermore, the efficiency of microorganisms in utilizing Fe appears to be strain-specific (Berman-Frank et al., 2007). Recent advances in metagenomic techniques have provided insight into the metabolic potential of several cultivated diazotrophs. For example, the recently sequenced genomes of *Trichodesmium erythraeum* IMS101 and *Crocosphaera watsonii* WH8501 indicate that these diazotrophs have significant differences in their capabilities to acquire and use different species of dissolved organic P (DOP) (Dyhrman and Haley, 2006; Dyhrman et al., 2006). It follows that in natural populations of diazotrophs, the availability of Fe and P will have different impacts on N_2_ fixation based on the diazotrophic taxa present.

This study extends the findings of Goebel et al. (2010) by investigating the spatial variability of *nifH* expression associated with the same natural populations of cyanobacterial diazotrophs using reverse transcription (RT)-qPCR assays. BNF rates associated with the small size-fraction (<10 μm) of these natural populations were also measured using ^15^N_2_ tracer assays (Montoya et al., 1996). To investigate the nutrient limitations of diazotrophy in this ocean basin, five trace-metal clean bottle incubations were conducted over 36–48 h periods, with additions of Fe, inorganic phosphorus, and a combination of both (Fe+P). Both qPCR and RT-qPCR assays were used to determine changes in *nifH*-based abundances and *nifH* expression in five cyanobacterial phylotypes in response to these experimental conditions, and BNF rates for the small size-fraction were also measured. This study is one of the first attempts to use targeted functional gene expression to investigate the response to nutrient additions in natural assemblages of marine microorganisms.

## Materials and methods

### Cruise track, CTD and diel sampling

**Figure 1:**
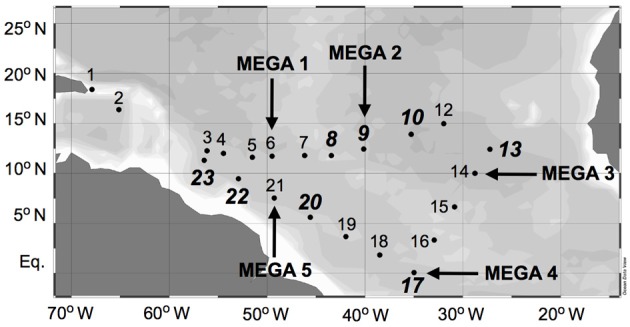
**Cruise track of SJ0609 aboard R/V *Seward Johnson* in September 2006**. Diel *nifH* expression is reported from stations italicized in bold. MEGA nutrient addition experiments were performed at stations designated with arrows.

Samples were collected during a 2006 Trans-Atlantic cruise aboard the R/V *Seward Johnson* (Figure 1). The eastbound leg of the cruise began in Barbados, and transited southeast into Amazon plume-influenced waters, then east to the Cape Verde Islands. The westbound leg of the cruise transited southwest from the Cape Verde Islands to the Equator, then transited northwest through Amazon plume-influenced waters again, back to Barbados. Samples were collected for the quantification of daytime and nighttime *nifH* expression in natural cyanobacterial populations, and for the corresponding N_2_ fixation rates, using Niskin bottles mounted in a rosette coupled to a conductivity temperature depth (CTD) instrument from the surface (between 5–15 m), the deep chlorophyll max (DCM; between 65–125 m depth), and depth of the oxygen minimum (between 150–200 m). Seawater from Niskin bottles was collected into HCl-cleaned polycarbonate bottles with HCl-cleaned tubing. If the CTD was deployed within 2 h of local noon or midnight, samples were collected directly from the Niskin bottles, and immediately filtered and frozen (as described below). In some cases, this was not possible, and the water was collected into 4 L bottles and incubated at surface seawater temperatures with shading to approximate the appropriate light intensity until harvested at local noon/midnight. All samples for molecular analyses were filtered using a Masterflex peristaltic pump (Cole Parmer, Vernon Hills, IL) and size-fractionated onto a 25 mm diameter 10 μm pore-size polyester filter (GE Osmotics, Minnetonka, MN) and a 25 mm 0.2 μm Supor filter (Paul Corp., Port Washington, NY), held in parallel swinnex filter holders (Millipore, Billarica, MA). Filters for RNA samples were transferred into microcentrifuge tubes containing 0.1 and 0.5 mm diameter glass beads (BioSpec Products, Bartsville, OK) and 400 μL RLT buffer (Qiagen, Germantown, MD, USA) with 1% (v/v) betamercaptoethanol (BME), immediately frozen in liquid N_2_, and kept at −80°C until extraction. Filters for DNA samples were also flash frozen in liquid N_2_ after being transferred in to microcentrifuge tubes containing 500 μL TE buffer and glass beads and stored at −80°C. Samples were also taken and analyzed for inorganic nutrient concentrations (nitrate+nitrite, phosphate, and silicate) and chlorophyll *a*, and the methods used for sampling and analysis along with the resulting data was reported in Goebel et al. (2010).

### Nutrient enrichment (MEGA) experimental set-ups

At stations 6, 9, 14, 17, and 21, large volumes of surface water were collected for nutrient enrichment experiments (called the “MEGA” experiments). These MEGA experiments were conducted using trace metal clean techniques throughout the entire sampling process. The seawater, pumped from approximately 10 m using acid-washed tubing, was collected directly into large acid-washed mixing carboys within a laminar-flow trace metal clean working area. For each of the five MEGA experiments, the seawater was subsequently dispensed into 4-L acid-washed polycarbonate bottles. The 4 L bottles were designated for sampling at various time intervals (0, 12, 24, 36, and 48 hours) and with different nutrient amendments (control, Fe, P, and Fe+P). For MEGA experiments 1–4, Fe bottles were amended with FeCl_3_ (dissolved in 0.01 N HCl) for a final concentration of 10 nM of added Fe and P bottles were amended with KH_2_PO_4_ (stock solution previously eluted through a Chelex-100 column to remove trace metals) for a final concentration of 200 nM of added PO_4_. As the Amazon River plume influenced nutrient levels at Station 21, final concentrations of Fe and P were increased to 50 nM and 2 μM, respectively, in MEGA5. Time zero bottles were sampled immediately. The remaining bottles were placed in spectrally corrected blue deck-board incubators that were continuously flushed with surface seawater to maintain the proper temperature until ready for sample processing. Samples for RNA extraction and ^15^N_2_ rate measurements were collected from duplicate bottles at each time point and for each type of nutrient enrichment; samples for DNA extraction were collected from triplicate bottles. DNA and RNA samples were filtered (500–2000 mL depending on time point) as described above. RNA samples were collected at every time point whereas DNA samples were only collected at 0, 24, and 48 h.

### ^15^N_2_ rate measurements

Rates of N_2_ fixation were measured using tracer additions of ^15^N_2_ gas following the general protocol of Montoya et al. (1996). In brief, incubation bottles were filled to the point of overflowing, while carefully excluding bubbles, then 2 mL of ^15^N_2_ gas at 1 atm pressure was added using a gas-tight syringe. The bottles were incubated under simulated *in situ* conditions in a flowing seawater incubator on deck for 12, 24, 36, or 48 h, and were terminated by gentle pressure filtration through a 10 μm Nitex screen and a precombusted GF/F filter (small size fraction material). Material collected on the 10 μm screens (large size fraction material) was rinsed off and transferred to a precombusted GF/C filter. Filters were dried at 60°C on board the ship then stored over desiccant for analysis ashore.

All isotope measurements were carried out by continuous-flow isotope ratio mass spectrometry using a Micromass Optima interfaced to a CE Instruments NC2500 elemental analyzer for online combustion and purification of organic N_2_. All isotope abundances were corrected for instrument and blank effects as described in Montoya (2008), and rates were calculated using the mass balance approach of Montoya et al. (1996).

### Nucleic acid extractions and cDNA generation

DNA from bottle incubations was extracted using the modified DNeasy Plant kit (Qiagen) method described in Moisander et al. (2008) with the following modifications: the glass beads were added to autoclaved bead-beating tubes prior to sampling; freeze-thaw cycles used liquid N_2_ rather than an ethanol/dry ice slurry; and the final elution volume was 50 μL. DNA extracts were stored at −20°C. This is the same protocol used to process the complementary samples presented in Goebel et al. (2010), which are used to provide context for this study. DNA extracts were quantified using the Quant-iT™ PicoGreen® kit assay kit (Invitrogen, Carlsbad, CA, USA) and measured using a SpectraMax M2e spectrophotometer (Molecular Devices, Sunnyvale, CA, USA).

RNA extractions were performed using a modified RNeasy Plant Mini kit (Qiagen) protocol. To ensure that extractions were carried out in an RNase-free environment all surfaces and pipettors were cleaned with RNase Zap solution (Invitrogen). Prior to extractions, a DNAse I working solution was made from an RNase-free DNAse I stock (RNase-free DNase set; Qiagen), by adding 10 μL of the stock (2.73 units μL^−1^) to 70 μL Buffer RDD for each sample. Bead-beating tubes containing filters frozen RLT/BME were thawed on ice and agitated in a bead-beater twice for 2 min each, cooling on ice between bead-beatings (Mini-Beadbeater-96; Biospec Inc., Bartlesville, OK, USA). Filters were removed using sterile needles and discarded. Samples were then centrifuged for 2 min, and the supernatant was transferred into a new sterile 2-mL microfuge tube. 250 μL of 100% ethanol was added to the supernatant, and then samples were gently mixed by inversion and transferred onto RNeasy Mini spin columns. Spin columns were centrifuged for 15 s, and the flow through was discarded. 350 μL of RW1 buffer was then added to each spin column, and after another centrifugation step for 15 s the flow through was discarded. DNA was removed using an on-column DNase digestion step where 80 μL of the DNAse I working solution was added directly to the spin column and incubated at room temperature for 1 h. After DNase digestion, an additional 350 μL of buffer RWI was added to the spin column, followed by another centrifugation step for 15 s. RNA retained on the column was cleaned with two consecutive additions of 500 μL RPE buffer and centrifugation for 15 s followed by an additional 2-min centrifugation to dry columns. RNA was eluted into sterile 1.5 mL microfuge tubes by adding 50 μL RNase-free water to the column, letting sit at room temperature for 1 min and then centrifuging for 2 min. All centrifugation steps were carried out at 8000 × *g*. RNA extracts were quantified using the Quant-it™ RiboGreen® RNA assay kit (Invitrogen), according to manufacturer's guidelines. RNA extracts were stored at −80°C. Complementary DNA (cDNA) was generated from RNA extracts via RT using the SuperScript™ III First Strand Synthesis System for RT-qPCR (Invitrogen) as described for RT-qPCR reactions in Turk et al. (2011).

### Quantitative PCR assays for abundance (qPCR) and expression (RT-qPCR)

Quantitative PCR (qPCR) and RT quantitative PCR (RT-qPCR) were used to quantify *nifH* gene copies and *nifH* transcripts, respectively, from five different cyanobacterial phylotypes that have been detected in the TNAtl (Langlois et al., 2008; Goebel et al., 2010; Turk et al., 2011) described in Table 1.

**Table 1:** **Cyanobacterial *nifH* qPCR and RT-qPCR targets**.

**Cyanobacterial target**	**References**	**Genbank accession no. of standard**	**Efficiency (E)**	**Size fraction**
UCYN-A	Church et al., 2005	AF059642	102%	0.2–10 μm
UCYN-B	Moisander et al., 2010	DQ481411	88%	0.2–10 μm
*Trichodesmium*	Church et al., 2005	DQ404414	94%	>10 μm
*Richelia* in *R. clevi* (RR)	Church et al., 2005	DQ225757	98%	>10 μm
*Richelia* in *H. haucki*i (HR)	Foster et al., 2007	DQ225753	98%	>10 μm

The efficiency (E) of each assay was determined using the formula E = 10^−1/m^ − 1, where m is the slope from the linear regression applied to standards with 10^0^–10^7^ gene copies reaction^−1^. All DNA and RNA samples were size fractionated, and the target size fraction for each phylotype is indicated.

Abbreviations: UCYN-A uncultivated unicellular cyanobacteria group A UCYN-B uncultivated unicellular cyanobacteria group B.

All qPCR reactions used reaction conditions, plate set-up, instrumentation, thermocycling parameters, and approach to calculating *nifH* transcripts/gene copies L^−1^ according to methods described in Goebel et al. (2010).

Taking into consideration the dilutions made during the RT reactions, the volume of nucleic acid extractions, and the volume of seawater filtered, the limit of detection (LOD) and limit of quantitation (LOQ) for the RT-qPCR reactions in this study were between 32–63 and 250–500 *nifH* transcripts L^−1^ seawater, respectively. The LODs and LOQs were slightly lower (13–25 and 100–200 *nifH* copies L^−1^) in the qPCR analysis of DNA extracts. For both qPCR and RT-qPCR analyses, samples were designated as “detected not quantified” (DNQ) where the detected signal was greater than the LOD, but fell below the LOQ.

For cDNA samples, each RT and no-RT sample was screened for inhibition using the UCYN-A primer/probe set by spiking the reaction with a 10^5^ standard and determining the percent efficiency. No inhibition was observed. For a majority of the samples, no-RTs did not amplify, indicating that the DNase step successfully removed all DNA present. In the several samples where amplification was observed in the no-RTs, the *nifH* transcripts reaction^−1^ were always in the range of DNQs, and were subtracted from the *nifH* transcripts reaction^−1^ calculated for the RT amplification.

In order to discuss *nifH* expression per N_2_-fixing cell in natural diazotrophic populations, the following assumptions were made: DNQs were estimated to be 100 *nifH* copies L^−1^ for DNA and 250 *nifH* copies L^−1^ for RNA; UCYN-A, -B, and *Trichodesmium* were assumed to have one gene per N_2_-fixing cell; and the RR phylotype was assumed to have four vegetative cells per heterocyst, and the HR phylotype was assumed to have three vegetative cells per heterocyst (Foster and Zehr, 2006).

Where possible, a Student's *t*-test (homoscedastic, 2-tailed) was used to determine the statistical significance of differences between control and treatment incubations (*p* < 0.05) in both abundance (qPCR) and expression (RT-qPCR) data from the MEGA experiments. It is important to note, however, conditions of normality cannot be verified with replicates, and that in many cases, expression and/or abundance data in control incubations were either UD or DNQ, which represents real information that cannot be included in a *t*-test.

## Results

### Nitrogenase gene expression in natural populations of cyanobacteria in the tropical north atlantic

**Figure 2:**
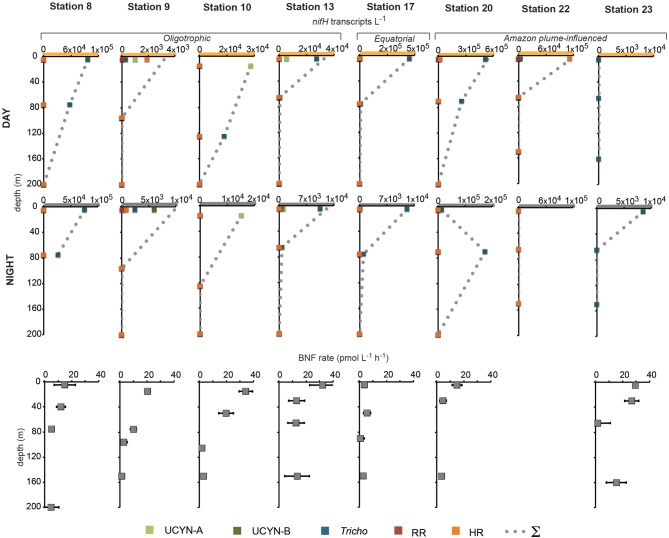
**Daytime and nighttime *nifH* expression and BNF rates associated with the <10 μm size-fraction in natural populations of diazotrophs**. No BNF rates were measured at Station 22. Note the different scales on the x-axis of *nifH* expression data, and the y-axis for BNF rate depths. Abbreviations: UCYN-A, uncultivated unicellular cyanobacteria group A; UCYN-B, uncultivated unicellular cyanobacteria group B; *Tricho*, *Trichodesmium*; RR, *Richelia* in *Rhizosolenia* (Het-1); HR, *Richelia* in *Hemiaulus* (Het-2); and Σ, sum of all *nifH* transcripts L^−1^ for both size-fractions quantified at each station and depth.

**Figure 3:**
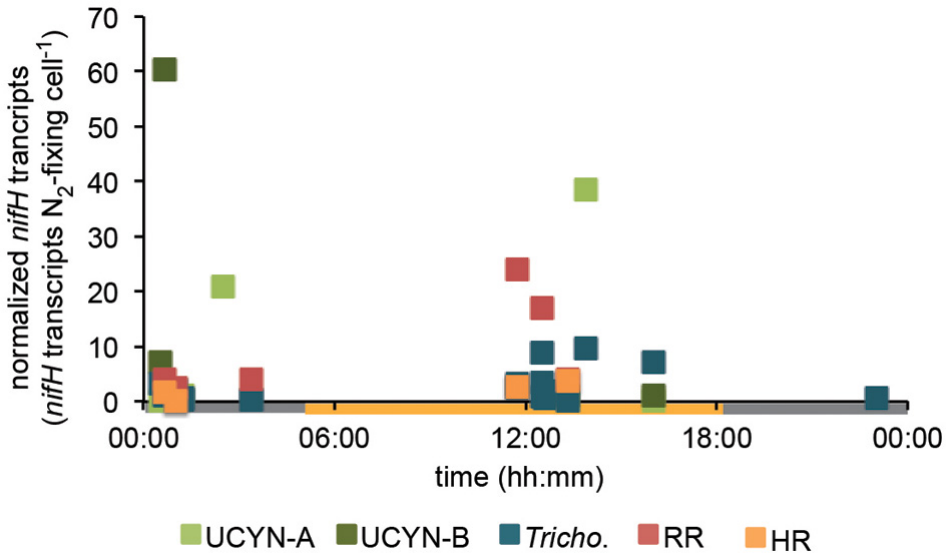
**Diel *nifH* expression normalized to the abundance of each phylotype**. Expression data across all stations was pooled, and transcript numbers were normalized to each N_2_-fixing cell using *nifH*-based abundances at each station reported in Goebel et al. (2010), and assumptions outlined in the methods section. DNQs for DNA and RNA were changed to 100 and 250 *nifH* copies/transcripts L^−1^ for these calculations, respectively.

In order to investigate the diel *nifH* expression in natural populations of cyanobacterial diazotrophs in the TNAtl, we used a combination of direct sampling from the CTD and shipboard incubations at eight stations along the SJ0609 transit (Figure 1). These stations spanned a range of environmental conditions (Table 2), from waters with low salinities (<35 ppt) resulting from the Amazon River plume (Stations 20, 22, and 23), to the oligotrophic waters surrounding the Cape Verde Islands (Stations 10 and 13), and equatorial waters with detectable *NO*_3_+NO_2_ and PO_4_ at the surface, characteristic of equatorial upwelling (Station 17). Diel *nifH* expression was determined for UCYN-A, UCYN-B, *Trichodesmium*, HR and RR using RT-qPCR assays. In addition to considering the absolute *nifH* transcripts L^−1^ for each phylotype at three depths in the photic zone (Figure 2), daytime and nighttime expression data was also normalized to represent the number of transcripts associated with each N_2_-fixing cell (Figure 3), which had been used as a proxy for which diazotrophs are most actively expressing *nifH* (Zehr et al., 2007). This required dividing the *nifH* transcript copies L^−1^ by the *nifH* gene copies L^−1^ reported by Goebel et al. (2010), which were determined from complementary sampling efforts at these stations, and the assumptions outlined in the method section for the ratio of vegetative and heterocystous cells in the HR and RR. In general, the highest *nifH* expression for the unicellular cyanobacterial phylotypes UCYN-A and UCYN-B was measured in the eastern part of the basin (Stations 9, 10, and 13), and highest *nifH* expression for the heterocystous cyanobacterial symbionts was measured along the southern cruise track in the western part of the basin (Station 22). *Trichodesmium nifH* expression was measured at all eight stations (Figure 2).

**Table 2:** **SJ0609 station information, environmental and experimental parameters for all stations and depths sampled for both diel investigations and MEGA experiments**.

**Station**	**Lat. (ddm)**	**Long. (ddm)**	**depth (m)**	**Temp (°C)**	**Salinity (psu)**	**chl *a* (μg L^−1^)**	**NO_3_ + NO_2_ (μM)**	**PO_4_ (μM)**	**Si(OH)_4_ (μM)**	**Time of ^15^N_2_ gas injection in BNF rate incubations (hh:mm)**
**6**	**11.712**	**−49.486**	**5**	**27.5**	**36.1**	**0.13**	**bd**	**bd**	**bd**	**na**
8	11.787	−43.447	5	27.4	36.1	0.11	0.01	bd	bd	03:15
			75	25.5	36.3	0.42	0.10	bd	0.01	
			200	13.1	35.5	bd	nm	nm	nm	
**9**	**12.434**	**−40.135**	**5**	**26.4**	**36.4**	**nm**	**bd**	**bd**	**bd**	14:50/09:20–13:27^*^
			96	22.4	36.8	0.47	0.08	0.04	0.01	
			200	13.3	35.5	nm	24.42	1.43	7.78	
10	13.921	−35.284	15	26.0	36.4	0.02	bd	bd	bd	04:30
			125	21.0	37.0	0.12	3.57	0.11	bd	
			200	15.7	36.0	0.01	19.61	0.86	3.30	
13	12.413	−27.247	5	26.9	36.2	0.07	0.01	bd	bd	16:55
			65	21.7	35.9	0.43	0.04	bd	1.13	
			200	12.2	35.3	bd	24.61	1.54	9.26	
**14**	**10.000**	**−28.772**	**5**	**27.2**	**36.0**	**nm**	**0.05**	**bd**	**0.01**	12:00–15:30^*^
**17**	**0.081**	**−34.991**	**5**	**26.8**	**36.2**	**0.20**	**0.48**	**0.58**	**bd**	04:20/13:06–00:48^*^
			75	25.5	36.5	0.37	1.26	0.59	bd	
			200	13.3	35.3	nm	14.11	1.27	8.61	
20	5.601	−45.598	5	28.6	33.0	0.30	0.15	0.13	5.23	03:15
			70	26.7	36.3	0.62	0.14	0.10	0.07	
			150	12.7	35.4	0.01	19.74	1.42	8.49	
**21**	**7.531**	**−49.260**	**5**	**28.8**	**32.8**	**0.24**	**nm**	**nm**	**nm**	12:51–15:30^*^
22	9.467	−52.93	5	28.3	34.7	nm	nm	nm	nm	na
			73	25.0	36.6	nm	1.18	0.48	1.75	
			150	15.6	35.9	nm	0.29	1.37	6.52	
23	11.303	−56.443	5	28.4	32.9	nm	nm	nm	nm	00:10
			76	26.9	36.2	nm	nm	nm	nm	
			160	20.5	36.8	nm	nm	nm	nm	

Stations where MEGA experiments were conducted are in shaded rows with bold text, and the time of ^15^N_2_ gas tracer injection for associated BNF rate measurements is marked with an asterisk (

* *)*.

Abbreviations na not applicable nm not measured bd below detection limit.

UCYN-A was found to be actively expressing *nifH* around mid-day at a depth of 15 m at Station 10, with 39 *nifH* transcripts N_2_-fixing cell^−1^, and also had elevated levels of expression at night at this station and depth (Figure 2). Although UCYN-B was detected in DNA samples in surface waters at Station 10 (Goebel et al., 2010), expression of *nifH* from UCYN-A accounted for all of the characterized transcripts in both the daytime and nighttime samples. The observation of *nifH* expression during the day is consistent with what we now know about the photoheterotrophic metabolism of UCYN-A (Zehr et al., 2008; Tripp et al., 2010). However, measuring high transcript numbers during the night is contrary to diel observations made at station ALOHA, where *nifH* expression was not observed in the dark (Zehr et al., 2007).

UCYN-B *nifH* expression was observed exclusively at night in surface waters at Station 9 (5 m) and at the DCM at Station 13 (65 m). This supports previous observations, in both cultures and in the environment, that UCYN-B temporally separates N_2_ fixation to protect the nitrogenase from oxygen evolved during oxygenic photosynthesis (Church et al., 2005; Zehr et al., 2007; Shi et al., 8501). Although *nifH*-based abundances are generally considered a proxy for the dominant diazotrophs in a given sample, such assumptions can be incorrect when comparing *nifH*-based abundance data with *nifH* transcript data. This is evident in surface samples from Station 9, where no UCYN-B was detected using *nifH*-based abundances (implying UCYN-B abundance was <63 *nifH* copies L^−1^, the LOD for this qPCR assay; Goebel et al., 2010), yet *nifH* transcripts from UCYN-B were the predominant contributor to the overall *nifH* pool characterized at 5 m (Figure 2), even representing the highest normalized *nifH* transcripts N_2_-fixing cell^−1^ among all the samples and diazotrophs characterized in this study (Figure 3).

*Trichodesmium* was the most abundant phylotype across this ocean basin (Goebel et al., 2010) and *nifH* transcripts were consistently high, with the notable exception of having low copy numbers and expression in surface waters at Station 10. However, when transcripts were normalized to *nifH* gene copies (reported from complementary samples in Goebel et al. (2010)), normalized transcripts from *Trichodesmium* were consistently low (Figure 3), less than 10 *nifH* transcripts N_2_-fixing cell^−1^. The highest normalized *nifH* expression measurements were found midday at Station 10 (125 m), Station 13 (5 m), and Station 17 (5 m). At station ALOHA, normalized gene expression for *Trichodesmium* has been measured to be an order of magnitude greater (~100 transcripts gene^−1^; Zehr et al., 2007).

As described for UCYN-B, *nifH*-based abundances (from DNA samples) do not always predict *Trichodesmium* nitrogenase activity. For example, at Station 13, it appeared that UCYN-A was the dominant diazotroph in surface waters using *nifH*-based abundances (4.6 × 10^4^ *nifH* copies L^−1^; Goebel et al., 2010). However, it appears that *Trichodesmium* may be the most active N_2_-fixing organism, as a majority of the *nifH* transcripts quantified from these surface waters were from *Trichodesmium* (2.4 × 10^4^ *Trichodesmium nifH* transcripts L^−1^, compared to 4.8 × 10^3^ UCYN-A *nifH* transcripts L^−1^; Figure 2).

RR *nifH* expression was highest at midday in surface waters at Station 20 at 3.6 × 10^3^ *nifH* transcripts L^−1^. Despite being two-orders of magnitude lower than *Trichodesmium nifH* transcripts, normalized *nifH* transcripts N_2_-fixing cell^−1^ in this sample were the highest measured in this study for non-unicellular cyanobacterial diazotrophs (24 *nifH* transcripts N_2_-fixing cell^−1^; Figure 3). This indicates that although RR may be less abundant than *Trichodesmium*, its contribution to the fixed N pool in this region may be just as important, if not more important, than *Trichodesmium*. RR and *Trichodesmium* were the only two phylotypes with detectable expression in the warm equatorial waters at Station 17 (4.2 × 10^2^ and 4.3 × 10^5^ *nifH* transcripts L^−1^, respectively). RR was either not detected or had low abundances at all other stations and depths. However, it should be noted that RR abundances and expressions were not measured for Stations 22 and 23, a region of the Atlantic basin heavily influenced by the Amazon plume where abundant RR and HR have previously been documented (Foster et al., 2007).

HR *nifH* transcripts were detected in both daytime and nighttime surface samples at Stations 9 and 20 and in the daytime surface sample at Station 22. At Stations 9 and 20, the normalized transcript abundance was low, at <4 *nifH* transcripts N_2_-fixing cell^−1^. At Station 9, *nifH* transcripts from HR accounted for a majority of the transcript pool for the daytime sample (Figure 2), despite being lower in abundance than UCYN-A (1.5 × 10^3^ and 5.6 × 10^3^ *nifH* copies L^−1^ for HR and UYCN-A respectively; Goebel et al., 2010). It is important to note that the highest measured *nifH* transcripts for HR were in surface waters at Station 22 (1.1 × 10^5^ *nifH* transcripts L^−1^), but no DNA was analyzed for this sample, so the normalized expression cannot be determined. Both RR and HR *nifH* expression are positively correlated with temperature (*r*^2^ = 0.27, *p* = 0.01 and *r*^2^ = 0.20, *p* = 0.03, respectively), and negatively correlated with salinity (*r*^2^ = 0.18, *p* = 0.04 and *r*^2^ = 0.26, *p* = 0.01, respectively), but these correlations, though significant, are weak. In the case of RR, these results contradict those reported in Foster et al. (2009) from a region farther south (the eastern equatorial Atlantic), where the correlation between RR and temperature was negative.

### Biological nitrogen fixation rates in the tropical north atlantic

BNF rates associated with the small size-fraction were generally highest in surface waters at the oligotrophic stations and reached their highest rates at the easternmost Stations 10 and 13, at 34.3 ± 5.0 pmol L^−1^ h^−1^ (15 m), and 32.3 ± 5.0 pmol L^−1^ h^−1^ (5 m), respectively. They were also comparably high at Station 23 (26.7 ± 5.3 pmol L^−1^ h^−1^; 30 m) where no *nifH* expression from UCYN-A or UCYN-B was quantified (Figure 2). BNF rates were lowest throughout the photic zone at the equatorial Station 17 (between 0.6 and 5.7 pmol L^−1^ h^−1^). At most stations, the biomass collected in the large size fraction (>10 μm) was too small to provide a robust measurement of isotope content and N_2_-fixation activity, therefore, only the small-size fraction data is reported here.

The overall UCYN-A *nifH* expression does show a significant positive correlation with integrated BNF rates associated with the small size-fraction (*r*^2^ = 0.33, *p* = 0.0005). However, HR *nifH* expression showed the strongest correlation with integrated BNF rates (*r*^2^ = 0.40 and *p* = 0.002), across all the stations sampled. This correlation between a diatom-diazotroph association (DDA) and integrated BNF rates assumed to be associated with much smaller cells is surprising and difficult to interpret given that the *nifH* expression was quantified from the 10 μm filter, and was not measured on the 0.2 μm filter, which is a common practice for these DDAs. There have been several reports of free-living planktonic heterocystous cyanobacteria (e.g., Gómez et al., 2005) that might contribute to BNF rates in this size-fraction, but it is assumed that the *Richelia* phylotype targeted by this qPCR assay lives within the host diatom frustule. This correlation might also result from either the physical disruption of the HR association during filtration, or the rapid incorporation and excretion of ^15^N by HR in the 10 μm size fraction, followed up by assimilation into particulate N of smaller microorganisms, an effect which has been reported for *Trichodesmium* (Mulholland et al., 2006). Due to the technical approaches used to measure BNF rates in this and other studies, discussed in detail below, the values reported here are likely underestimates.

### Nitrogenase gene expression in response to nutrient amendment experiments

Sets of five trace-metal clean bottle incubations, called the “MEGA” experiments, were conducted at select stations (Figure 1) to investigate nutrient limitations of diazotrophy in major cyanobacterial phylotypes across the TNAtl Ocean basin. Three experiments were conducted on diazotrophic communities in oligotrophic waters (MEGA1—Station 6; MEGA2—Station 9; and MEGA3—Station 14), one in equatorial water with evidence of upwelling (MEGA4—Station 17) and one from the lower salinity, higher-nutrient Amazon-plume influenced waters (MEGA5—Station 21).

Results from each experiment will be discussed in detail below; however, some general findings were consistent across all experiments. Enhanced *nifH* expression in cyanobacteria associated with the small size-fraction (UCYN-A and UCYN-B) rarely correlated with a stimulation of BNF rates in these experiments, which were carried out over a relatively short time period (between 36–48 h), as the intent was to capture the first order changes evident in gene expression. Despite this lack in response, BNF rates measured in controls from MEGA experiments were almost always higher than those measured for the *in situ* communities discussed above. It is also important to note that an increase in population size for any given diazotroph throughout the duration of these experiments (as inferred from an increase in *nifH* gene copies L^−1^ in DNA extracts) was rare, even in cases where stimulation of *nifH* expression with respect to the control was measured. Finally, diazotroph *nifH*-based abundances from time zero samples correlate well with those reported at the same station in Goebel et al. (2010).

#### MEGA1

**Figure 4:**
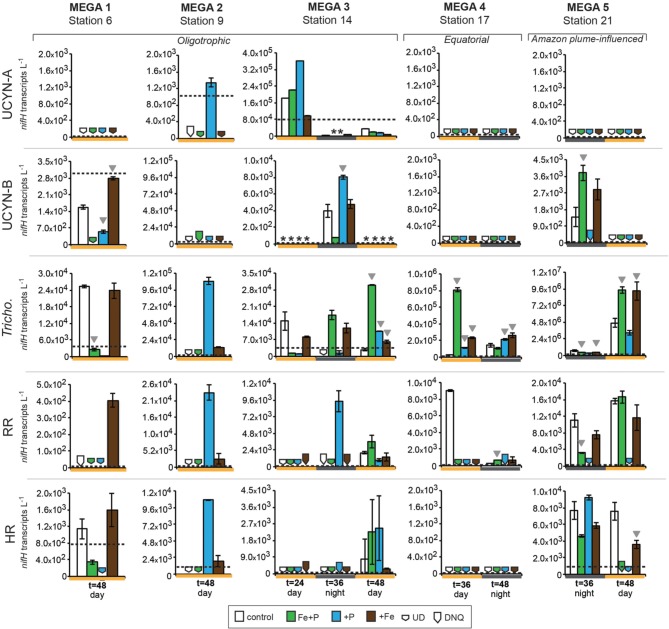
**Expression of *nifH* in response to nutrient additions in the MEGA experiments**. Quantified *nifH* transcripts L^−1^ in time zero samples in each experiment for each phylotype is marked with a dotted line. UCYN-A and UCYN-B *nifH* transcripts L^−1^ were quantified from 0.2 μm filters; *Tricho*, RR, and HR *nifH* transcripts L^−1^ were quantified from 10 μm filters. Error bars indicate the standard error, where possible. Significant differences between control and treatment (Student's *t*-test, *p* < 0.05) are marked with a grey triangle. Missing data is marked with an asterisk (^*^). Note the different scales used for the y-axis. Abbreviations: UD, undetected; DNQ, detected not quantified.

**Figure 5:**
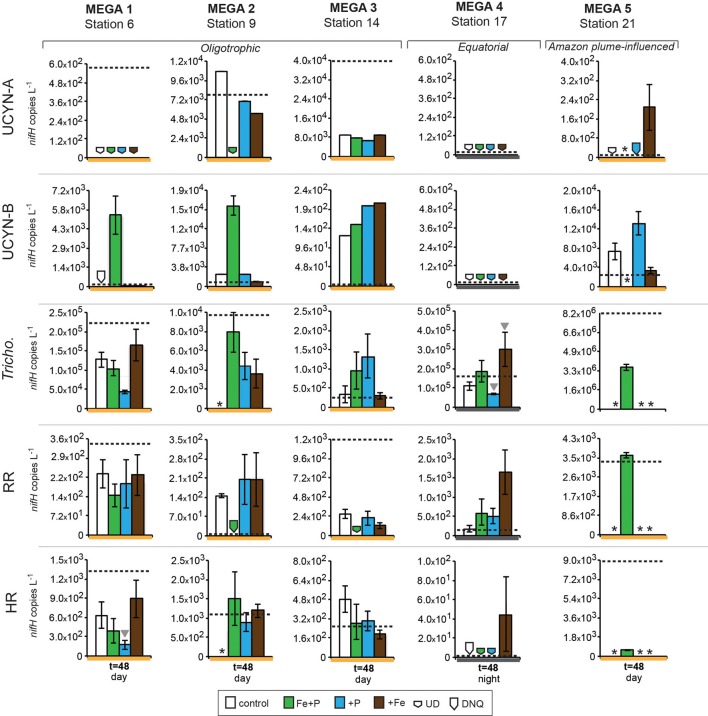
**Changes in *nifH*-based abundances in response to nutrient additions in the MEGA experiments between time zero and *t* = 48 h samplings**. Quantified *nifH* copies L^−1^ in time zero samples in each experiment for each phylotype is marked with a dotted line. UCYN-A and UCYN-B *nifH* copies L^−1^ were quantified from 0.2 μm filters; *Tricho*, RR, and HR *nifH* copies L^−1^ were quantified from 10 μm filters. Error bars indicate the standard error, where possible. Significant differences between control and treatment (Student's *t*-test, *p* < 0.05) are marked with a grey triangle. Missing data is marked with an asterisk (^*^). Note the different scales used for the y-axis. Abbreviations: UD, undetected; DNQ, detected not quantified.

Conducted at Station 6, where the low salinity lens from the Amazon River plume is no longer detected, the diazotrophic community in MEGA1 was dominated by *Trichodesmium* and HR based on the *nifH*-based abundances in time zero samples, and UCYN-B, *Trichodesmium* and HR were all showing signs of active regulation of the *nif* operon based on the detectable expression of *nifH* in time zero samples. UCYN-A was determined to be present at low abundances in time zero samples (Figure 5), but there was no detection of *nifH* expression at any point in the experiment, and based on the decrease in *nifH* abundances by *t* = 48 h, it appears that this small population of UCYN-A dwindled to below detection limits, or crashed all together. UCYN-B *nifH* expression appeared to be stimulated in the Fe treatment at *t* = 48 h, despite being present at low abundances in this treatment, which is somewhat surprising as *t* = 48 h was taken during the day (Figure 4), and without sampling at the peak of *nifH* nighttime expression it is difficult to interpret these results. Furthermore, UCYN-B *nifH*-based abundances increased in the Fe+P treatment, but it is impossible to determine if there were different factors driving UCYN-B growth and N_2_-fixing activity in this experiment. *Trichodesmium* was present at high abundances in the control and all three treatments (~10^5^ cells L^−1^; Figure 5) and was actively transcribing *nifH* at the onset of the experiment; however, none of the nutrient additions appeared to stimulate *nifH* expression (Figure 4). Although RR was not expressing *nifH* at the beginning of this experiment, elevated *nifH* expression, with respect to the *t* = 48 h control was measured in the Fe treatment alone, which implies that during the course of the experiment, RR began to regulate the *nif* operon in the presence of Fe. No BNF rates were available from this experiment.

#### MEGA2

**Figure 6:**
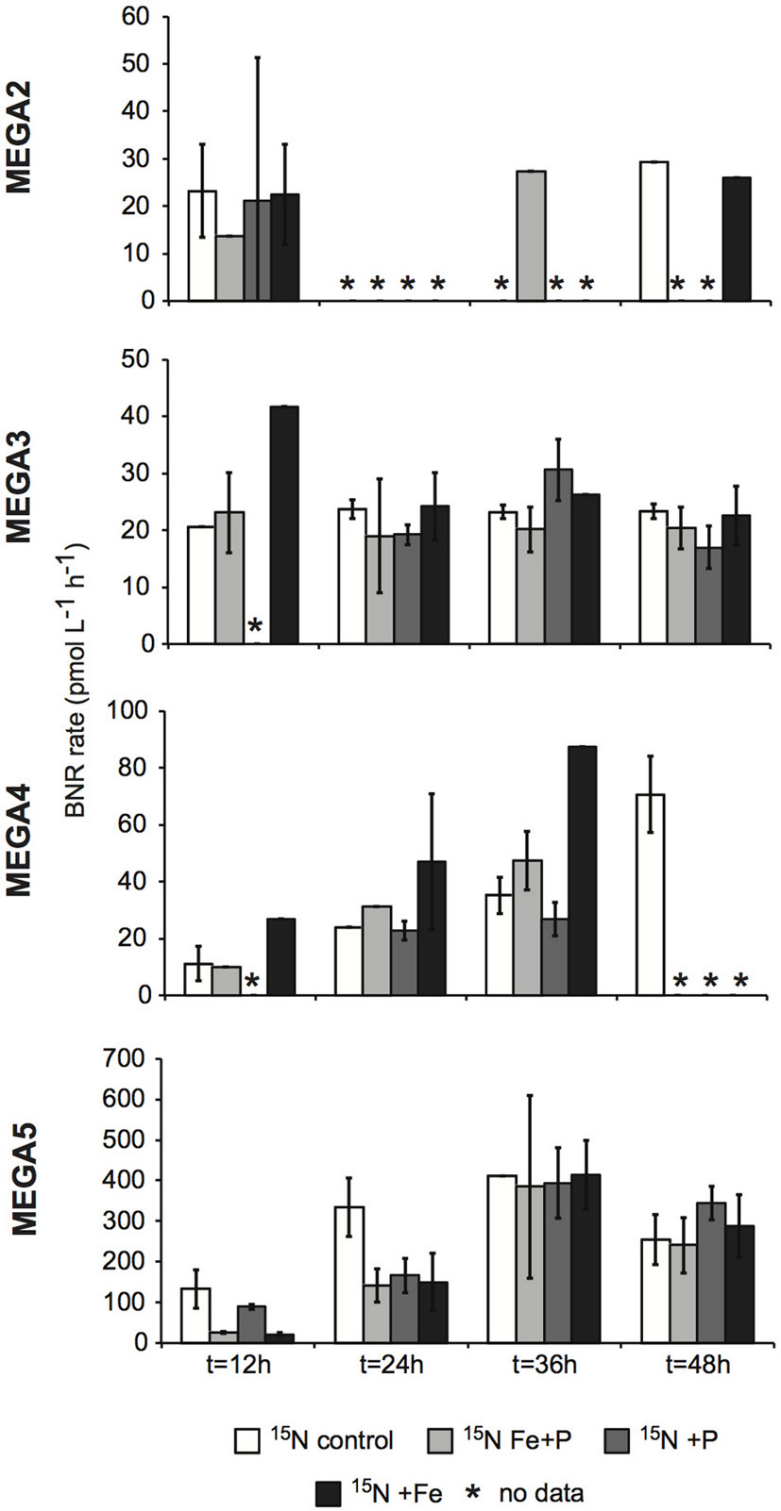
**Biological N_2_ fixation rates associated with the <10 μm size-fraction in response to nutrient additions in the MEGA experiments**. BNF rates were measured using the ^15^N_2_ tracer method. Error bars indicate the standard deviation for two replicates, where possible. Missing data is marked with an asterisk (^*^). Note the different scales used for the y-axis.

In MEGA 2, conducted at Station 9, the diazotrophic community was comprised predominantly of UCYN-A, *Trichodesmium*, and HR (Figure 5; Goebel et al., 2010). Although RR was not detected in time zero samples, *nifH*-based abundances in *t* = 48 h samples indicate that they were present in low abundances in the P and Fe treatments as well as the control. UCYN-B was also detected at low abundances in time zero samples, but the Fe+P treatment stimulated a measurable increase in *nifH*-based abundances by *t* = 48 h, from 6.7 × 10^2^ *nifH* copies L^−1^ to 1.5 × 10^4^ *nifH* copies L^−1^. As with MEGA1, the sampling time at the end of the experiment occurred during the daytime, so no information about the stimulation of *nifH* expression in UCYN-B was obtained in this experiment. Expression of *nifH* in UCYN-A, *Trichodesmium*, HR and RR was stimulated in the P treatment, increasing by 10^3^–10^4^ *nifH* transcripts L^−1^ with respect to the control expression (which was UD or DNQ for all phylotypes; Figure 4). There was also an increase in *nifH* expression in the Fe treatments for *Trichodesmium*, HR and RR, but it was consistently about an order of magnitude less than the P response. BNF rates at *t* = 12 h did not show any measurable response to any of the three treatments, and rates associated with the *t* = 48 h samples were unavailable (Figure 6). As discussed in detail below, it is possible that a lack of measureable response in the first 12 h results from incomplete dissolution of the ^15^N_2_ tracer gas which was injected into BNF rate incubations during the daytime (Table 2).

#### MEGA3

In the easternmost region of the TNAtl, near the Cape Verde Islands, the prevalence of UCYN-A and UCYN-B has been reported (Langlois et al., 2008; Goebel et al., 2010; Turk et al., 2011), and it is hypothesized that these unicellular diazotrophs may contribute to a significant portion of the fixed N in this area. At Station 14, the source waters for the MEGA3 experiment, the most abundant diazotroph in time zero waters was UCYN-A at 4.0 × 10^4^ *nifH* gene copies L^−1^. UCYN-B was present below detection limits of the qPCR assay at time zero. RR was the second most abundant diazotroph at 1.2 × 10^3^ *nifH* copes L^−1^, which is in contrast to *nifH*-based abundances reported in Goebel et al. (2010), where RR was not detected at this station. *Trichodesmium* and HR were both present at approximately 10^2^ *nifH* copies, L^−1^. Only UCYN-A and *Trichodesmium* had detectable *nifH* expression in time zero samples. The *nifH* expression from unicellular diazotrophs was stimulated in P treatments in the *t* = 24 h daytime sampling (UCYN-A) and the *t* = 36 h nighttime sampling (UCYN-B). At the *t* = 48 h sampling however, very low numbers of *nifH* transcripts associated with UCYN-A were quantified, and no response to the treatments was observed. The opposite is true for *Trichodesmium*; no *nifH* expression response was quantified in the *t* = 24 h sampling, but in the *t* = 48 h sampling, a significant response in *nifH* expression was seen in all treatments (Figure 4, Fe, *p* = 0.04; P, *p* = 0.003, and Fe+P, *p* = 0.0002). This was accompanied by a decrease in UCYN-A *nifH*-based abundances in all treatments, and an increase in *Trichodesmium nifH*-based abundances in both the Fe+P and P treatments between time zero and *t* = 48 h samples. These results indicate that UCYN-A was able to respond more quickly to P additions than the other diazotrophs, and that in P-replete conditions, UCYN-A *nifH* transcription was stimulated more quickly than in *Trichodesmium*, HR and RR. BNF rates for MEGA3 were determined for *t* = 12 h (except P), *t* = 24 h, *t* = 36 h and *t* = 48 h (Figure 6). For a majority of the samples, no measurable difference in BNF was measured between the control and treatments. The only measurable stimulation in BNF rate was seen in the *t* = 36 h sample for the P treatment, which corresponds with the increased response in *nifH* transcription by UCYN-A and UCYN-B in the first 36 h of this experiment.

#### MEGA4

In the equatorial waters at Station 17, the diazotrophic community was composed almost entirely of *Trichodesmium* (Figure 5 and Goebel et al., 2010), although RR was also detected at low abundances in time zero samples (2.2 × 10^2^ *nifH* copies L^−1^). Neither *Trichodesmium* nor RR had detectable *nifH* expression at time zero, but this is likely due to the nighttime sampling, therefore it is not possible to determine whether these populations were actively regulating the *nif* operon at the onset of MEGA4. It is reasonable to assume, however, that the *Trichodesmium* was active, as *nifH* expression was measured in the natural population (Figure 2) at this station. Both the P and Fe additions stimulated small increases in *Trichodesmium nifH* transcripts L^−1^, but a much larger increase in *nifH* expression was seen in the Fe+P treatment (Figure 4), implying that N_2_ fixation by *Trichodesmium* in this region may be co-limited by both Fe and P. However, by the end of the incubation, *Trichodesmium* abundances were significantly higher in Fe treatments (*p* = 0.02; Figure 5). Even though unicellular diazotrophs were not detected at this station, BNF rates associated with the <10 μm fraction in MEGA4 were comparable, and in some cases higher, than the rates measured in MEGA3. Furthermore, there was a measurable stimulation of BNF rates associated with the Fe treatment in *t* = 36 h samples (Figure 6). There are several potential explanations for the measurement of these rates in the small size fraction, including the presence of unicellular cyanobacterial phylotypes or heterotrophic diazotrophs not targeted by these qPCR assays, or the incorporation of ^15^N by organisms in the small size fraction that was originally fixed then released as reduced N by *Trichodesmium*, which has been shown to occur on very short time scales (<12 h) in the Gulf of Mexico (Mulholland et al., 2006). It is also possible that measured rates in the small size fraction result from the disruption of *Trichodesmium* filaments during filtering and size fractionation. Previous studies have visually documented the breakage of *Trichodesmium* filaments in the filtration process (Letelier and Karl, 1996), an observation consistent with the reported detection of *Trichodesmium nifH* in the small size fraction using qPCR-based approaches (Zehr et al., 2007; Moisander et al., 2008). Goebel et al. (2010) conducted qPCR analysis of both size fractions on the natural population of *Trichodesmium* along this cruise track, and reported abundances as the combined total of both size fractions. In surface waters (5 m) at Station 17, 11% of the total *nifH* copies L^−1^ were found in the small size-fraction (5.4 × 10^3^ out of a total of 4.8 × 10^4^ *nifH* copies L^−1^; Table 3). If the detection of *Trichodesmium nifH* in the 0.2–10 μm fraction results from disrupted colonies that were fixing N at previously reported cell-specific rates (20 fmol N cell^−1^ h^−1^; Capone, 2001), and that the same disruption effect occurred in the filtration for rate measurements, it follows that the cells captured in this size fraction would appear to have incorporated ^15^N_2_ at a rate of approximately 100 pmol L^−1^ h^−1^, which could account for the measured rates (Table 3), despite this N actually being fixed in the >10 μm community.

**Table 3:** **Size-fractionated *Trichodesmium nifH* qPCR results from natural populations along this cruise track from 5 m DNA samples reported in (Goebel et al., 2010), and estimated BNF fixation rates associated with disrupted *Trichodesmium* colonies measured as part of the small size fraction**.

**Station**	***Tricho. nifH copies L^−1^***	***Tricho. nifH copies L^−1^***	***Tricho. nifH copies L^−1^***	**Estimated BNF rate (pmol L^−1^ d^−1^) of disrupted *Tricho*. in**
	**<10 μm fraction**	**>10 μm fraction**	**Σ**	**<10 μm fraction**
6	1.2E + 04	3.6E + 05	3.8E + 05	238
8	4.8E + 02	1.8E + 05	1.8E + 05	10
9	2.1E + 01	1.5E + 03	1.6E + 03	0
10^*^	0.0E + 00	1.6E + 03	1.6E + 03	0
13	2.9E + 02	3.1E + 03	3.4E + 03	6
14	1.0E + 00	2.1E + 02	2.1E + 02	0
17	5.4E + 03	4.3E + 04	4.8E + 04	107
20	1.4E + 03	2.0E + 05	2.0E + 05	29
21	6.1E + 04	1.1E + 06	1.2E + 06	1216
22	nm	nm	na	na
23	1.2E + 04	nm	1.2E + 04	240

The assumed cell specific N_2_ fixation rate is 20 fmol N cell^−1^ hr^−1^ (Capone, 2001), and it is assumed that single Trichodesmium filaments and colonies have the same cell-specific rates. Station 10 (

* ) data is reported from 30 m depth.

Abbreviations Tricho. Trichodesmium nm not measured na not applicable.

#### MEGA5

MEGA5 was the only nutrient amendment experiment conducted in Amazon Plume-influenced waters (Station 21). *Trichodesmium* dominated the diazotrophic community in this experiment (8.2 × 10^6^ *nifH* copies L^−1^), although UCYN-B, RR and HR were also present in abundances on the order of 10^3^ *nifH* copies L^−1^ (Figure 5). UCYN-A was not detected in qPCR assays at time zero, but was present in the Fe treatment at *t* = 48 h, however, no UCYN-A *nifH* transcripts were detected in this experiment. Both UCYN-B and *Trichodesmium nifH* expression was stimulated with respect to control treatments in Fe+P and Fe treatments (Figure 4). Nutrient amendments did not appear to stimulate *nifH* expression in RR or HR. Although stimulation of UCYN-B *nifH* expression in Fe and Fe+P treatments was not reflected in the BNF rates, it is notable that the highest BNF rates in this study were measured from this experiment, reaching as high as 400 pmol L^−1^ h^−1^ (Figure 6). As described above for MEGA4, based on the high abundances of *Trichodesmium* in time zero samples, the most likely explanation is that colonies or single *Trichodesmium* filaments were disrupted during filtration and captured on the GF/F filter as part of the small size fraction. Estimated BNF rates based on the *Trichodesmium nifH* qPCR analysis of the small size-fraction in the natural population of *Trichodesmium* (Goebel et al., 2010), using assumptions described above and in Table 3, support this hypothesis.

## Discussion

### Diel *nifH* gene expression and BNF rates in natural populations of cyanobacteria

This is the first study to quantify daytime and nighttime *nifH* expression of key cyanobacterial phylotypes in the natural populations of diazotrophs in the TNAtl. This builds upon, and is complementary to, the *nifH* gene-based abundances for these same phylotypes reported by Goebel et al. (2010). Gene expression provides information on the activity or physiological status of cells, since detection of transcripts implies active gene transcription. Although the expression of *nifH* cannot be directly linked to N-limitation or active N_2_ fixation, transcripts are not long-lived, thus detection of *nifH* transcripts indicates the organisms are alive, and that they are regulating expression of the nitrogenase operon.

*Trichodesmium nifH* transcripts comprised a majority of the total *nifH* transcript pool quantified in this study, and were the most abundant transcript (by many orders of magnitude) measured at five out of the eight stations (Figure 2). This study provides further evidence that *Trichodesmium* is the most widespread diazotroph in this ocean basin. However, another important outcome of this study is that *nifH* gene-based abundances do not always predict which diazotroph is actively transcribing *nifH* (and presumably fixing N_2_), as discussed above.

*Trichodesmium* consistently had low normalized *nifH* transcripts (<10 *nifH* transcripts N_2_-fixing cell^−1^; Figure 3), and it is unclear whether this reflects the TNAtl *Trichodesmium* population, or is an artifact of sampling times or extraction techniques. The ratio of *Trichodesmium nifH* transcripts to *nifH* gene-based abundances was also generally low in a study conducted south of this study site in the East Equatorial Atlantic (Foster et al., 2007), which utilized similar DNA/RNA extraction, cDNA generation and RT-qPCR techniques. In contrast, in two studies from the North Pacific Subtropical Gyre, normalized *nifH* transcripts were measured as high as 10^2^–10^3^ *nifH* transcripts per *nifH* gene copy (Church et al., 2005; Zehr et al., 2007). These two studies used a different DNA extraction technique, making direct comparisons to this study difficult. More importantly, however, the diel sampling resolution in Church et al. (2005) was much greater, and it is clear that in the North Pacific Subtropical Gyre, normalized *nifH* transcripts are >10 *nifH* cDNA copies gene copy^−1^ roughly between 04:00 and noon (local time), and vary over a 72-h period. A more high-resolution diel sampling scheme with TNAtl populations is ultimately needed to inform our interpretations, but it is reasonable to assume that that the peak of *nifH* transcription in *Trichodesmium* occurred in the early morning and was missed due to the sampling scheme.

BNF rates associated with the <10 μm size-fraction are not commonly measured. Using complementary methods in the South Pacific, Moisander et al. (2010) reported maximum BNF rates associated with environmental samples dominated by UCYN-A and UCYN-B of 4500 pmol L^−1^ h^−1^, and 26 pmol L^−1^ h^−1^, respectively. Kitajima et al. (2009) also measured significantly higher rates associated with the small size-fraction in the Western North Pacific (between 42–541 pmol L^−1^ h^−1^), but these numbers were acquired using the acetylene reduction assay, thus are considered gross N_2_ fixation rates, rather than net N_2_ fixation rates, like those measured by the ^15^N_2_ gas tracer method.

There are two important sources of underestimation in the BNF measurements reported in this and other studies. First, the amount of ^15^N incorporated into particulate N in the small size fraction was determined using the material captured on a GF/F filter, after being prefiltered using a 10 μm filter. Therefore the BNF rates reported are associated with organisms between 0.7 μm and 10 μm, while the *nifH* abundance and expression data includes microorganisms as small as 0.2 μm. Our best estimates for the diameter of a UCYN-A cell is 0.62 ± 0.17 μm (Thompson et al., 2012) and there is no reason to assume that the UCYN-A present in this study remained attached to it's recently identified picoeukaryote host (see “discussion” below), therefore it is likely that some of the ^15^N-labeled particluate N fixed by UCYN-A passed through the GF/F filter. Despite this discrepency, using GF/F filters remains a standard technique in this field, even in studies that endeavor to compare these measurements to the abundance of unicellular diazotrophs (e.g., Bonnet et al., 2009; Sohm et al., 2011; Großkopf et al., 2012; Halm et al., 2012).

The second source of underestimation is evidenced in a series of recent studies that report a significant and variable underestimation of BNF rates measured using the ^15^N_2_ gas tracer method resulting from the slow dissolution of gaseous ^15^N_2_ into the water phase (Mohr et al., 2010; Großkopf et al., 2012; Wilson et al., 2012). According to Mohr et al. (2010), based on results from experiments using *Crocosphaera* cultures, the most extreme underestimation of BNF rates using the ^15^N_2_ gas tracer technique occurs if the spike of ^15^N_2_ gas is initiated just as an organism is beginning to fix N_2_. Thus, ^15^N_2_ gas introduction in the early morning may most egregiously underestimate BNF rates associated with *Trichodesmium*, UCYN-A, HR, and RR which fix N_2_ during daylight hours while BNF rates associated with UCYN-B will be most compromised by ^15^N_2_ gas introduction in the late evening. Furthermore, there is some evidence that in natural communities where unicellular cyanobacteria or heterotrophs are the most abundant diazotrophs, the degree of underestimation of BNF rates is far greater than when *Trichodesmium* is the most abundant diazotroph, possibly as a result of different buoyancies (Großkopf et al., 2012).

The current study was conducted prior to the findings of Mohr et al. (2010), and without performing parallel bubble and dissolved seawater incubations, such as those in Wilson et al. (2012) and Großkopf et al., (2012), it is impossible to address the degree to which the BNF rates reported in this study are underestimated. However, it is reasonable to assume that BNF rates measured at Stations 9 and 10, where unicellular cyanobacterial transcripts dominated the *nifH* expression (Figure 2), are most heavily underestimated. At Stations 9 and 10, ^15^N_2_ gas introduction in the rate incubations occurred at 14:50 and 04:30, respectively (Table 2). Therefore, at both stations the timing of the gas introduction likely resulted in a significant underestimate of the BNF rate associated with UCYN-B (Station 9) and UCYN-A (Station 10), which may have impacted the overall BNF rate, presented here as an hourly average over a 24 h period.

### MEGA experiments indicate that Fe and P limitations on diazotrophy are spatially heterogeneous and phylotype-specific

The MEGA experiments are among the first to investigate the response of a natural assemblage of marine microorganisms to the addition of limiting nutrients by quantifying changes in functional gene expression (using RT-qPCR). Similar experiments have been conducted to study the response of diazotrophs to inorganic P additions in the oligotrophic North Pacific (Zehr et al., 2007) and the Great Barrier Reef (Hewson et al., 2007), but this is the first study to focus on *nifH* expression in the diazotrophic population in the TNAtl, and to use trace-metal clean techniques so that Fe responses could be investigated. Although there are several recent examples of using high throughput sequencing or microarray techniques to analyze the (meta)transcriptional responses of communities of marine microorganisms to experimental manipulations (e.g., Smith et al., 2010; Shi et al., 2011), these approaches are currently limited in the qualitative nature of their data.

It is important to note that one underlying assumption of the MEGA experiments is that the diazotrophs were actively regulating the *nif* operon—an assumption that is supported by the measurement of *nifH* transcripts in the natural diazotroph assemblage (discussed above) and in time zero samples. Although 48 h incubations may not be long enough to regularly see shifts in diazotroph abundances (via qPCR) or stimulation of BNF rates, it is assumed that the changes in *nifH* expression (via RT-qPCR) that might result from P, Fe, or Fe+P additions are possible because the community is N-limited overall and that diazotrophs are active but might be experiencing either P- or Fe-stress. Conversely, measuring no response in *nifH* expression during the course of a treatment might ultimately mean that the phylotype was not N-limited at the time, in addition to not being P- and/or Fe-limited.

The MEGA experiments revealed a distinct spatial pattern of *nifH* expression response to Fe, P, and Fe+P amendments, with Fe limitation of *nifH* expression in the western basin of the TNAtl, and P limitation of the unicellular diazotrophs in the eastern basin. This appears to contradict the findings of Sohm et al. (2008), who reported the quickest turnover of the P pool in the western part of the basin, which is considered a proxy for P-limitation. This contradiction may be reflective of the differences between nutrient limitation for the microbial community as a whole and that of a specific taxonomic group, like the diazotrophs. In addition to this spatial trend, there appear to be phylotype-specific responses, which strongly suggests that P and Fe (co-)limitation of diazotrophy is extremely heterogeneous on short time scales. Furthermore, over the time scale of these experiments, stimulation of BNF rates associated with the <10 μm size fraction and increases in diazotroph abundances were rarely measured, implying that diazotroph abundances are slow to respond to changing nutrient conditions. It is important to note however, that the above discussion of underestimation in these rate measurements due to the use of GF/F filters and the ^15^N_2_ gas tracer method, are applicable to the MEGA experiments as well. A summary of the MEGA experimental results can be found in Table 4.

**Table 4:** **Synthesis of MEGA experimental results, indicating the nutrient stress conditions that can be inferred**.

	**MEGA1**				**MEGA2**				**MEGA3**				**MEGA4**				**MEGA5**			
	**Present^a^**	**Active N-fix^b^**	**Fe-response^c^**	**P-response^d^**	**Present^a^**	**Active N-fix^b^**	**Fe-response^c^**	**P-response^d^**	**Present^a^**	**Active N-fix^b^**	**Fe-response^c^**	**P-response^d^**	**Present^a^**	**Active N-fix^b^**	**Fe-response^c^**	**P-response^d^**	**Present^a^**	**Active N-fix^b^**	**Fe-response^c^**	**P-response^d^**
UCYN-A	+	nd	nd	nd	+	**+**	−	**+**	+	**+**	−	**+**	−	nd	nd	nd	−	nd	nd	nd
UCYN-B	−	**+**	**+^e^**	−	+	**+**	na^e^	na^e^	−	na^e^	−	**+**	−	nd	nd	nd	+	na^e^	**+**	−
*Tricho*.	+	**+**	−	−	+	**+**	**+**	**+**	+	**+**	**+**	**+**	+	**+**	**+**	**+**	+	−	**+**	−
RR	+	−	**+**	−	−	−	**+**	**+**	+	−	**+**	**+^f^**	+	−	**+**	−	+	−	−	−
HR	+	**+**	−	−	+	**+**	**+**	**+**	+	−	−	**+**	−	nd	nd	nd	+	**+**	−	**+**

“+” *Indicates that the phylotype was present or exhibiting the specified response;* “−” *Indicates that the phylotype was not present or no response was detected.*

Abbreviations nd nifH expression not detected na not applicable (UCYN-B nifH expression during the day).

a Detection of nifH from phylotype in DNA (qPCR) at time zero.

b Detection of expression of nifH from phylotype (RT-qPCR) at time zero.

c Increased expression of nifH from phylotype (RT-qPCR) at time zero with respect to the control upon addition of Fe.

d Increased expression of nifH from phylotype (RT-qPCR) at time zero with respect to the control upon addition of P.

e Sample collected during the day.

f Expression response at night.

#### nifH expression responses to Fe/P additions in unicellular diazotrophs UCYN-A and UCYN-B

One of the most unexpected results of these nutrient amendment experiments is that increases of *nifH* expression in UCYN-A were observed only in P treatments. Fe additions did not stimulate the expression of *nifH* in UCYN-A, despite having stimulatory effects for other diazotrophs in the same samples (e.g., *Trichodesmium* in MEGA2 and MEGA3; Figure 4). There were detectable (0.74 ± 0.01 nM) concentrations of Fe in surface waters at nearby Station 13 at the time of MEGA3 (Goebel et al., 2010), and it is reasonable to assume that UCYN-A was able to fulfill its Fe demands with this ambient Fe in the first 24 h. Nonetheless, these findings raise interesting questions about the specific Fe demands and acquisition strategies of UCYN-A, especially given that *Trichodesmium* appears to be co-limited by Fe and P in this experiment (see “discussion” below). The two experiments where UCYN-A *nifH* expression response to P treatments (MEGA2 and MEGA3) were conducted in waters where concentrations of soluble reactive P (SRP) were low (<25 nM), while DOP was much higher (>200 nM) (Sohm et al., 2008), which may play a role in the apparent P-limitation of UCYN-A N_2_ fixation in these waters. Analysis of the recently sequenced genome of UCYN-A (Tripp et al., 2010) revealed that UCYN-A lacks important genes required for the hydrolysis of organic phosphates (compounds with a C-O-P bond) and utilization of phosphonates (compounds with a C-P bond), such as alkaline phosphatase (*phoA*), and genes in the phosphonate lyase pathway (*phnC-P*), therefore, based on our current understanding of this organism, it appears unlikely that UCYN-A is able to directly utilize components of the DOP pool. However, there is now evidence that UCYN-A forms a mutualistic association with a unicellular picoeukaryote prymnesiophyte (Thompson et al., 2012), and it is now unclear whether UCYN-A has a free-living state, thus the potential relevance of UCYN-As metabolic strategies (or lack there-of) is complicated by the paucity of information about the nature of this association, and the Fe and P acquisition strategies and requirements of its prymnesiophyte partner. In spite of these unknowns, observations from MEGA3 support the possibility that the UCYN-A/prymnesiophyte symbiosis, may have a competitive advantage in low SRP water, and may be able to outcompete *Trichodesmium*, RR and HR for inputs of inorganic P in the short term.

In MEGA1 and MEGA5, both conducted in the western part of the TNAtl basin, there was no indication that P availability had an impact on nitrogenase activity in UCYN-B. In contrast, UCYN-B *nifH* expression appeared to be responsive solely to the addition of Fe (Figure 4). At Station 21 (MEGA5), due to the influence of the Amazon River plume, the concentration of SRP was ~60 nM, which was among the highest measured concentrations by Sohm et al. (2008) during this cruise. It therefore seems likely that high SRP concentrations in surface waters were fulfilling the P demands of UCYN-B, driving N_2_ fixation in UCYN-B to Fe-limitation (despite concentrations of Fe in surface waters of 1.89 ± 0.03 nM; Goebel et al., 2010), which was relieved upon the addition of Fe.

It is less evident what factors might play a role in the stimulation of UCYN-B *nifH* expression by Fe at Station 6 (MEGA1), but Sohm et al. (2008) did report high concentrations of DOP along the northern leg of this cruise track, including Station 6. Although little is known about the exact chemical composition of the DOP pool, it is likely comprised partially of phosphomonoesters. Based on observations made from the genome analysis of a cultivated strain of UCYN-B, *Crocosphaera watsonii* WH8501, it is clear that at least some strains of UCYN-B have the genetic capability to utilize these compounds as a P source (Dyhrman and Haley, 2006), which may explain why *nifH* expression in UCYN-B responded to Fe, not P additions in MEGA1. It is important to note that this Fe stimulation was seen in samples taken during the daytime, and UCYN-B is known transcribe *nifH* at highest rates during the night.

However, like UCYN-A, *nifH* expression in UCYN-B is stimulated by the addition of P, and not Fe, in the easternmost experiment (MEGA3), despite comparable concentrations of DOP in these waters. This stimulation of *nifH* expression in UCYN-A and UCYN-B is reflected in the BNF rates measured in the small size-fraction, where a small increase time-averaged over the first 36 h is measured (Figure 6). It is possible that the differences in UCYN-B response between Station 6 and Station 14 are a result of different DOP pool compositions, or even strain differences in UCYN-B across the ocean basin not detected using the *nifH* qPCR assay, which broadly targets cultivated *Crocosphaera* strains and many environmental phylotypes characterized using degenerate *nifH* PCRs, but may not capture all phylotypes.

Although it was rare to observe *nifH*-based abundances of these diazotrophs increase throughout the duration of these experiments, UCYN-B increased in population size between time zero and *t* = 36/48 h samplings in all of the MEGA experiments in which it was detected (MEGA1–3 and MEGA5; Figure 5). In some experiments, growth was seen in different treatments than those where *nifH* expression was stimulated (e.g., MEGA5, Figures 4, 5) or where no *nifH* expression was observed at all (e.g., MEGA2, Figures 4, 5). The observation of decoupled growth and *nifH* expression might be explained by UCYN-B preferentially growing on other N sources, and/or natural grazing of UCYN-B being suppressed with respect to the other diazotrophs.

#### nifH expression responses to Fe/P additions in Trichodesmium

Like UCYN-B, *nifH* expression in *Trichodesmium* exhibited distinct spatial patterns in response to Fe, P, and Fe+P additions. In the Amazon River plume influenced experiment, MEGA5, *nifH* expression was stimulated in Fe and Fe+P treatments, while at Station 9 (MEGA2) the most prominent response in *nifH* expression was seen in the P treatments. Furthermore, *Trichodemsium* exhibited potential co-limitation of N_2_ fixation in the eastern (MEGA3) and in equatorial waters (MEGA4).

This is the first study that directly documents the Fe-limitation of N_2_ fixation activity in natural populations of *Trichodesmium* in the Amazon River plume using molecular techniques to our knowledge. Work by Webb et al. (2007) in the western Atlantic, showed stimulation of N_2_ fixation by the addition of dissolved inorganic P, not Fe. Together with the lack of evidence of extreme Fe deficiency (as evidenced by the production of an Fe deficiency protein, *IdiA*), they concluded that P-limitation of diazotrophy was in effect in this region. However, this study also documented heterogeneity in *Trichodemsium* P-stress responses, potentially explained by observations that P-stress was rarely observed in colonies with “tuft” morphologies.

As discussed above, results from MEGA3 indicate that there was a delay in *Trichodesmium nifH* expression response to the input of inorganic P, as *nifH* expression at the *t* = 24 h sampling was lower in the three treatments than in the control. In the *t* = 48 h sampling, however, response was seen in all three treatments, with the greatest response in the Fe+P treatment (Figure 4). This may be, in part, a function of the ability of *Trichodesmium* colonies to physically acquire P, which have a much larger cell to volume ratio and diffusive boundary layer thickness than UCYN-A/prymnesiophyte symbiont cells, which may drive diffusion limitation of nutrient acquisition, an effect that has been demonstrated to be an underlying factor in phytoplankton size distributions in the oligotrophic ocean (Chisholm, 1992).

*Trichodesmium* was the dominant diazotroph in the only equatorial upwelling waters sampled (Station 17); it was present at relatively high abundances at time zero samples in MEGA4 (1.7 × 10^5^ *nifH* copies L^−1^), and active diel expression was measured in corresponding samples (Figure 3). However, *Trichodesmium nifH* expression showed a significant response to all treatments (Fe, *p* = 0.002; P, *p* = 0.004, and Fe+P, *p* = 0.001), and appeared to be co-limited by P and Fe at this station as well (Figure 4). Inorganic P concentrations were high in surface waters at Station 17 (Table 2; and Sohm et al., 2008) due to upwelling, so the fact that *Trichodesmium nifH* expression was stimulated in the P and Fe+P treatment is unexpected, although it is important to note that the *nifH*-based abundances imply that growth was limited by Fe availability (Figure 5).

#### nifH expression responses to Fe/P additions in RR and HR

Despite the presence of RR in four of the five MEGA experiments (as determined via *nifH* qPCR at time zero; Figure 5), the data available indicate that it was not actively regulating the *nif* operon at the onset of most of these experiments. MEGA1 is the only experiment where no inferences can be made about the starting condition of the RR population, as time zero samples were taken at night, and no complementary data is available for the diel *nifH* expression of RR at this station. Over the course of these experiments, however, the expression of *nifH* was often measured in some or all of the treatments, indicating that RR began to transcribe the *nif* operon in the bottles. In Amazon Plume influenced waters (MEGA5), the RR population begins to express *nifH* in some of the treatments, but did not appear to be limited by Fe or P. In MEGA1, this onset of *nifH* expression was coupled to Fe-stress, and to P-stress in MEGA2. Like *Trichodesmium*, there is some indication that RR might be experiencing co-limitation of Fe+P in MEGA 3, as Fe and P additions alone did not result in an increase in *nifH* expression. In contrast, the natural population of HR was more likely to actively transcribing *nifH* than RR, as evidenced by detection of *nifH* samples in time zero samples, and in the natural diazotoph assemblage at Station 9 and Station 22 (Figures 4 and 2).

There is very little known about the Fe and P acquisition strategies and requirements of *Richelia* strains associated with either *R. clevi* or *H. hauckii*, and though these experiments provide some insight into the nutrient-stressed state of these populations in the TNAtl, further research is required to understand the metabolic potential of *Richelia* to use P-compounds, its Fe requirements, and the extent to which its diatom host impacts these requirements.

## Conclusions

This study is the first to report the daytime and nighttime *nifH* expression in natural populations of diazotrophs in the TNAtl, and for the most part, the dominant diazotrophs characterized by Goebel et al. (2010) were predictive of which organisms were actively regulating the *nif* operon at each of the stations measured. However, it is important to note that there were exceptions, and in those cases, DNA-based approaches alone would have not predicted which phylotypes were actively expressing *nifH*, thus likely fixing N_2_.

This is also one of the first studies to quantify the expression of a target gene in response to nutrient amendments within complex microbial assemblages in the marine environment. These findings indicate that gene expression can be used as a measure of changes in the activity of a community, especially over short time-scales where changes in biomass are not expected. In the TNAtl, the MEGA experiments not only revealed that cyanobacterial diazotrophs within a single community can sometimes respond differently to the same nutrient amendment, which emphasizes the importance of the metabolic capabilities of the individual organisms, but also that diazotrophs in the western region of the TNAtl responded primarily to Fe additions, while responding primarily to P additions in eastern waters. Further research is required to understand whether this phenomena exists year round, or whether it is heavily influenced by the seasonality of the Amazon River flow, to what extent these diazotrophs are actually experiencing P or Fe limitation and/or starvation, and to what extent the availability of Fe and different P compounds drive the diazotrophic community structure in the TNAtl. However, these findings underscore the importance of understanding the diazotroph community structure when considering whether a region is Fe or P limited.

### Conflict of interest statement

The authors declare that the research was conducted in the absence of any commercial or financial relationships that could be construed as a potential conflict of interest.
